# Age- and sex-specific differences in myocardial sympathetic tone and left ventricular remodeling following myocardial injury

**DOI:** 10.1186/s13293-024-00673-5

**Published:** 2025-01-16

**Authors:** Achi Haider, Susan Bengs, Angela Portmann, Sandro Fröhlich, Dominik Etter, Monika Maredziak, Geoffrey I. Warnock, Alexander Akhmedov, Sebastian Kozerke, Claudia Keller, Fabrizio Montecucco, Bruno Weber, Linjing Mu, Ronny R. Buechel, Vera Regitz-Zagrosek, Philipp A. Kaufmann, Giovanni G. Camici, Simon M. Ametamey, Catherine Gebhard

**Affiliations:** 1 Department of Nuclear Medicine, University Hospital Zurich, Zurich, CH-8091 Switzerland; 2 Center for Molecular Cardiology, University of Zurich, Schlieren, CH-8952 Switzerland; 3 Department of Radiology, Division of Nuclear Medicine and Molecular Imaging Massachusetts General Hospital, Harvard Medical School, Boston, MA 02114 USA; 4 Institute for Biomedical Engineering, University and ETH Zurich, Zurich, CH-8092 Switzerland; 5 Institute of Pharmaceutical Sciences, ETH Zurich, Zurich, CH-8093 Switzerland; 6 First Clinic of Internal Medicine, Department of Internal Medicine, University of Genoa, 6 viale Benedetto XV, Genoa, 16132 Italy; 7 IRCCS Ospedale Policlinico San Martino Genoa, Italian Cardiovascular Network, Genoa, 16132 Italy; 8 Institute of Pharmacology and Toxicology, University of Zurich, Zurich, CH-8057 Switzerland; 9 Institute for Gender in Medicine, Charité Universitaetsmedizin Berlin, Berlin, D-10115 Germany; 10 Department of Cardiology, Bern University Hospital, Inselspital, Freiburgstrasse 20, Bern, 3010 Switzerland

**Keywords:** Myocardial infarction, Sex hormones, Sex, [^11^C]meta-hydroxyephedrine, Positron emission tomography (PET), Cardiac magnetic resonance (CMR) imaging

## Abstract

**Background:**

Presentations and outcomes of acute myocardial infarction (MI) differ between women and men, with the worst outcomes being reported in younger women. Mental stress induced ischemia and sympathetic activation have been suggested to play a prominent role in the pathogenesis of MI in younger women, however, the impact of sex hormones on these parameters remains unknown.

**Methods:**

The effect of sex hormones and age on myocardial infarct size and myocardial sympathetic activity (MSA) was assessed in male and female, as well as young (4–6 months) and aged (20–22 months) FVB/N mice (*n* = 106, 60 gonadectomized and 46 sham-operated animals) who underwent in vivo [^11^C]meta-hydroxyephedrine ([^11^C]mHED) positron emission tomography (PET) and cardiac magnetic resonance (CMR) imaging 24 h after a 30 min myocardial ischemic injury.

**Results:**

MSA and catecholamine levels following myocardial injury were highest in young males (*p* = 0.008 and *p* = 0.043 vs. young females, respectively) and were reduced by orchiectomy. Accordingly, testosterone serum levels correlated positively with MSA (*r* = 0.66, *p* < 0.001). Males had a larger average infarct size and lower left ventricular contractility following myocardial injury than females (*p* < 0.05 vs. females). These sex differences were no longer evident in gonadectomized animals (p = NS vs. females). In female animals, estrogen depletion did not affect MSA (ovariectomy effect, *p* = 0.892). Female animals showed an age-dependent increase in MSA (*p* = 0.011), which was absent in males.

**Conclusion:**

Testosterone associates with an increase in sympathetic tone, contributing to adverse cardiac remodeling following MI. Conversely, females maintain sympathetic integrity, independent of sex hormones. Our results suggest a biological advantage of female sex in post MI recovery. Further research is warranted to confirm these findings in humans.

**Supplementary Information:**

The online version contains supplementary material available at 10.1186/s13293-024-00673-5.

## Abstract (plain language summary)

Heart attacks affect men and women differently, with younger women often experiencing worse outcomes than men. One reason for this difference might be how stress and heart nerve activity are influenced by sex hormones, though this has not been well understood to date.

In this study, we investigated how sex hormones and age affect heart damage and nerve activity in male and female mice. We used advanced imaging techniques to look at the hearts of young and older mice, some of which had their sex organs removed to study the hormone effects.

Our results showed that young male mice had higher nerve activity and stress hormone levels after a heart attack compared to young female mice. Removing the male sex hormone (testosterone) reduced this activity, suggesting testosterone worsens heart damage. In contrast, removing female sex hormones (oestrogen and progesterone) did not affect nerve activity in female mice. Females also maintained better heart function after a heart attack, regardless of their hormones.

In summary, male sex hormones may worsen early heart attack recovery by increasing stress on the heart, while female hearts were more resilient in our study. This information could help refine risk stratification and treatment of heart attack in men and women.

**Supplementary Information:**

The online version contains supplementary material available at 10.1186/s13293-024-00673-5.

## Highlights

In a validated murine model of acute myocardial infarction (MI), young males show higher myocardial sympathetic activity (MSA) and stress hormone levels compared to their female counterparts.

Circulating testosterone levels correlated with an enhanced MSA post MI in males.

Female mice maintain better heart function after a heart attack, regardless of estrogen levels.

Sex differences in MI outcomes are eliminated when sex hormones are removed.

These findings highlight a potential protective role of female sex in post MI recovery.

**Supplementary Information:**

The online version contains supplementary material available at 10.1186/s13293-024-00673-5.

## Introduction

Cardiovascular disease (CVD) is the most common cause of death among both men and women. Although significant refinements in therapeutic strategies have led to an overall decline in CVD mortality, these advances have less benefited women than age-matched men [1]. Accordingly, current CVD mortality rates in Europe are higher in women than in men, accounting for 47% of deaths in women and 38% of deaths in men [1]. Furthermore, death rates in women presenting with acute myocardial infarction (MI) are higher than those in age-matched men [2, 3], despite women having less plaque burden and a lower rate of obstructive coronary artery disease [4]. Amongst all demographic groups, outcomes of acute MI are worst in younger women [5] in whom a worrisome increase in MI incidence and case fatality has been observed over the past decade [6–8]. This sex disparity in MI outcomes may be attributed to differences in pathophysiology and presentation [4], less guideline-directed care in women [9, 10], and diagnostic strategies being optimized for a male population [11]. There is also an ongoing debate on whether these differences result from sociocultural (‘Gender’) or biological (‘Sex’) differences between women and men [12].

Amongst biological mechanisms, the involvement of the brain-heart axis and the sympathetic nervous system in triggering sex differences in clinical outcomes of MI through downstream effects on autonomic, immune, and vascular physiology, has been considered [13]. Indeed, we recently reported a strong association between metabolic activity of the amygdala - a brain centre regulating negative emotions and stress - myocardial dysfunction, and subclinical inflammation in women, but not in men [14, 15]. Also, women, in particular younger women, with a recent MI perceive greater psychological stress, encounter a higher risk of mental-stress induced ischemia, and display higher sympathetic activity than their male counterparts [16, 17]. The latter persisted for approximately nine months after the index event and was associated with an unfavourable prognosis [16]. These observations led to the hypothesis that a relatively greater magnitude of sympathetic activation may predispose women to worse outcomes after MI. However, the impact of sex hormones – and their changes with age – on sympathetic activity following MI is currently unclear. In fact, while the association between mental stress and myocardial ischemia seems to be strongest in younger women [17], there is evidence suggesting an increased baseline sympathetic activity in older women [18]. In addition, previous work in this field is hampered by the use of unspecific markers of sympathetic outflow such as muscle sympathetic nerve activity (MSNA) or heart rate variability, which do not capture direct cardiac sympathetic tone. The latter can be quantified by myocardial [^11^C]meta-hydroxyephedrine ([^11^C]mHED) uptake obtained from positron emission tomography (PET) images, which is the current gold standard for the assessment of cardiac sympathetic activity in humans and was recently validated for its use in mouse models [19]. Thus, **the aim of this study** was to analyze the impact of sex hormones and age on cardiac sympathetic activity and infarct size in a murine model of ischemia/reperfusion injury.

## Methods

### Animals

FVB/N mice of both sexes were obtained from Janvier Labs (France). A total of 106 animals (51% females) underwent cardiac magnetic resonance (CMR) and PET imaging at the age of 4–6 (young cohort) or 20–22 (aged cohort) months. Animals were randomized into subgroups that were subjected to either sham surgery or gonadectomy (Gx) at the age of one month. Overall, there were eight groups (*n* = 106, 60 gonadectomized and 46 sham-operated animals) with the following number of animals in each group: young-sham females, *n* = 15; young-Gx females, *n* = 15; young-sham males, *n* = 13; young-Gx males, *n* = 16; old-sham females, *n* = 9; old-Gx females, *n* = 16; old-sham males, *n* = 9; old-Gx males, *n* = 13. All mice were subjected to ischemia/reperfusion injury either at the age of 4–6 months or at the age of 20–22 months. Following a 30 min ligation of the left anterior descending artery, animals were allowed to recover for 24 h before serial PET and CMR scanning was performed. Immediately after the MRI scan, animals were sacrificed, and blood/urine samples were harvested for sex hormone and catecholamine measurements, respectively. All animal experiments were approved by the Cantonal Veterinary Office of Zurich in Switzerland and were performed in accordance with the Swiss animal welfare laws under the license ZH207/2016. All animals were housed in individually ventilated cages with ad libitum access to water and food and under specific pathogen-free (SPF) conditions.

### Gonadectomy

General anesthesia was performed by intraperitoneal injection of a mixture containing 10 mg/kg ketamine, 5 mg/kg xylazine and 5 mg/kg carprofen. For ovariectomy, anesthetized female mice were placed in lateral decubitus position on a heating pad to maintain their body temperature. The incision area was cleaned and disinfected with an iodine solution. The incision was performed into the skin and muscle layers of the abdominal wall. The ovary was externalized, and the dissection was performed between the ovary and the uterus horn. Subsequently, the incision was closed with a sterile absorbable surgical vicryl thread and disinfected using iodine solution on a sterile compress. The procedure was performed for both ovaries. For castration of male mice, the outer scrotal wall was disinfected using an iodine solution, and access was obtained to the testes using a sterile scalpel blade. The testes were externalized, and the testicular cords were cauterized with a thermocautery device. Subsequently, the testes were pushed back to the inguinal canal, and the skin closed using sterile absorbable surgical vicryl thread. The incision area was disinfected using an iodine solution.

### Ischemia-Reperfusion (I/R) Injury

Mice received 0.1 mg/kg body weight buprenorphine (Temgesic, Indivior Eu Ltd, UK) subcutaneously 30 min prior to surgery. Animals were anaesthetized with 5% isoflurane in oxygen-enriched air and maintained at 2–4% isoflurane throughout the surgical intervention. Throat and ribcage were shaved, and the skin was disinfected with 80% EtOH. Animals were placed in supine position with paws taped to the operating table equipped with an integrated heating pad (Harvard Apparatus™ Homeothermic Blanket Systems with Flexible Probe, Fisher Scientific, USA). Temperature was monitored and controlled with a flexible thermistor probe. Midline cervical incision was performed, and underlying tissues were retracted to uncover the trachea. An endotracheal tube was inserted, and mechanical ventilation was applied with a tidal volume of 175 µL and 150 breaths/min. Anesthetic depth was monitored by clinical parameters, including respiration depth and rate of around 110 breaths/min, color of mucous membranes and inner organs, movement and reflexes. A left lateral thoracotomy was performed via the third intercostal space. The pericardial sac covering the heart was removed, followed by the ligation of the LAD using an 8 − 0 Ethilon suture (Polymed Medical Center AG, Switzerland). The ligation was secured with a polyethylene tube to prevent damage to the artery. After 30 min of ischemia, the LAD occlusion was released. Effective reperfusion was confirmed by visible color restoration of the ischemic tissue. Thoracic incision layers were closed using a 5 − 0 Prolene running suture (Polymed Medical Center AG, Switzerland) to adapt the ribs. The thorax was drained with a syringe removing air from the pleural space, followed by another 5 − 0 Prolene suture to close the skin. Intubation was removed as soon as the animal showed spontaneous respiration. Mice were re-administered 0.1 mg/kg buprenorphine within 4–6 h after surgery. Animals underwent serial PET and CMR imaging after a reperfusion period of 24 h.

### Cardiac sympathetic imaging by Positron Emission Tomography/Computed tomography (PET/CT)

**Fig. 1:**
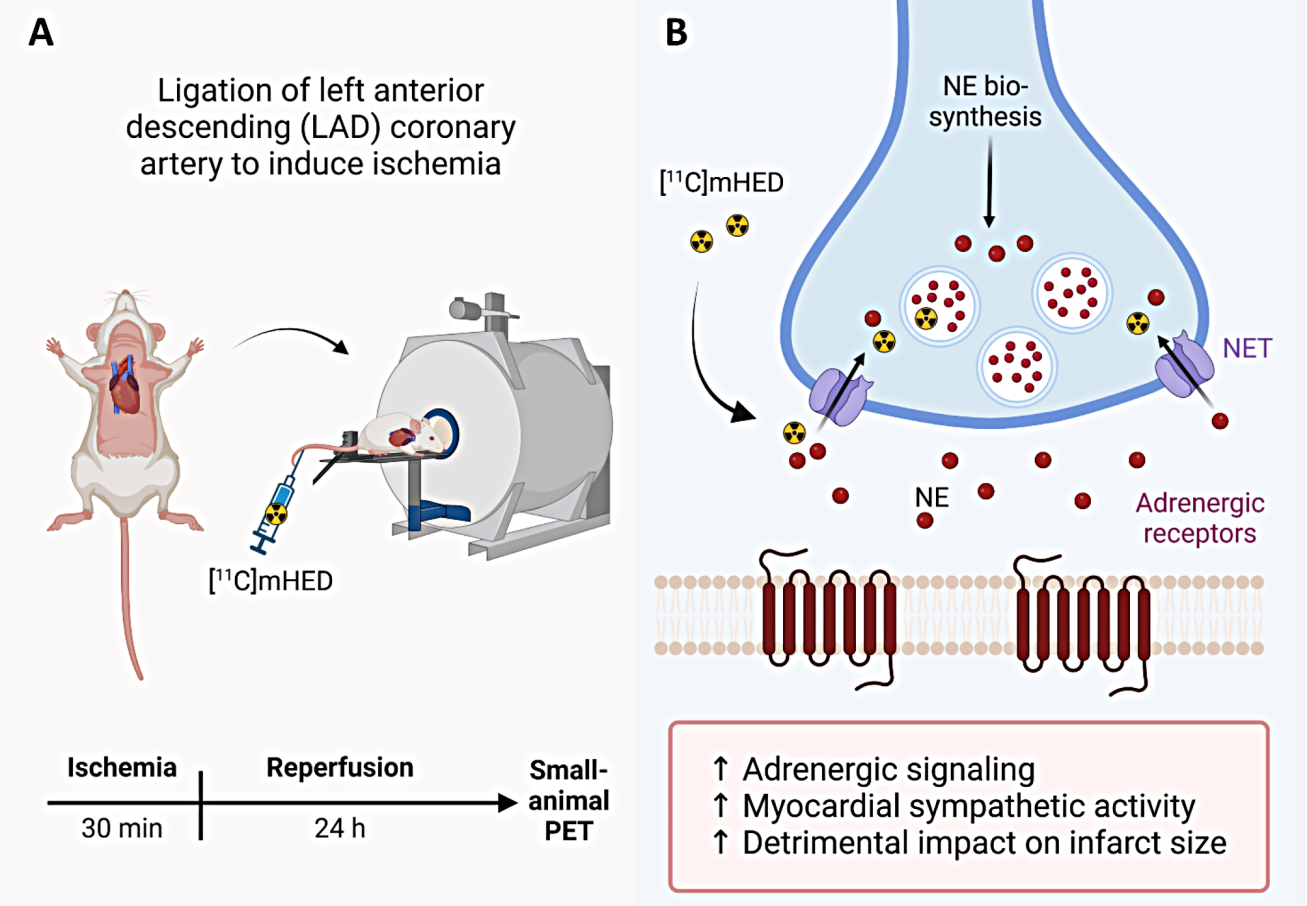
Myocardial sympathetic activity (MSA) assessed by [^11^C]mHED PET in male and female FVB/N mice after myocardial injury. **A**. Experimental design of the study with FVB/N mice subjected to 30 min ligation of the left anterior descending (LAD) artery, followed by a 24 h reperfusion period and subsequent PET imaging. **B**. Cardiac sympathetic nerve terminal and the suggested mechanism by which [^11^C]mHED is taken up via the norepinephrine reuptake transporter (NET) at sympathetic nerve terminals [19]. Abbreviations: [^11^C]mHED, [^11^C]meta-hydroxyephedrine; NE, norepinephrine; PET, positron emission tomography

An overview of the study design for the [^11^C]mHED study is provided in Fig. 1A. We and others have previously shown that [^11^C]mHED is taken up to pre-synapse via norepinephrine transporter 1 (NET) at sympathetic nerve terminals in mice, as depicted in Fig. 1B, and can be used to assess myocardial sympathetic integrity in rodents [19, 20]. PET/CT imaging was performed prior to CMR in animals anaesthetized with isoflurane (5% for induction and 2.0–4.0% for imaging) in oxygen-enriched air. Body temperature was monitored with a rectal thermistor as previously reported [21]. A bolus of 8.0-13.4 MBq [^11^C]mHED was injected in the tail-vein 1 min after the start of the scan. Radioactivity distribution was recorded with a Super Argus PET/CT tomography (Sedecal, Spain) in dynamic acquisition mode and over a time period of 61 min, followed by a CT scan for anatomical orientation and tissue attenuation correction as previously reported [22, 23]. PET data were reconstructed with a user-defined protocol at a voxel size of 1.16071_*_10^− 4^ cm^3^ and processed with PMOD v.3.8 (PMOD Technologies, Switzerland). A volume of interest (VOI) delineating the myocardium at the anterior midventricular wall was manually drawn. Myocardial [^11^C]mHED uptake was derived from the averaged frames of the initial 5–14 min post injection. Myocardial sympathetic activity (MSA) is presented as 1 – myocardial [^11^C]mHED uptake in % injected dose per mL (%ID/mL).

### Cardiac magnetic resonance imaging

CMR studies were conducted on a Bruker BioSpec 70/30 USR magnetic resonance scanner (Bruker BioSpin AG, Germany) operating at 5 MHz (7.0 Tesla), which was equipped with a Bruker console running on ParaVision 6.0.1 (Bruker Cooperation, USA) as well as ^1^H receive-only 2 × 2 mouse cardiac surface array coil (Bruker BioSpin AG, Switzerland). Mice were anaesthetized with isoflurane (4% for induction and 1.8–2.5% for imaging) in oxygen-enriched air, as previously reported [24]. Electrocardiogram electrodes were inserted subcutaneously into the paws and a pneumatic pillow sensor was immobilized on the abdominal region. Heart rate, respiration (breaths/min), and body temperature (°C) were recorded using SAII MR compatible monitoring and gating software (Small Animal Instruments Inc, USA). Body temperature was monitored with a rectal thermistor probe (ERT model 1030 control/gating module, Small Animal Instruments Inc, USA). A warm-water circuit was employed to maintain the temperature at 36–37 °C. Heart rates between 350 and 450 bpm and respiratory rates of 60–80 breaths/min were observed.

Initial heart localization and the acquisition of a four-chamber view was performed with a localizer and T1-FLASH scan. For the two-chamber view of the left ventricular (LV) long axis, a cine fast low angle shot (Cine-FLASH) with simultaneous respiratory self-gating (double gating) was carried out with the following parameters: Field of view = 25 mm x 25 mm, matrix dimension = 192 × 192, slice thickness = 0.8 mm, flip angle = 15, repetition time = 8 ms, echo time = 2.4 ms, number of averages = 8 resulting in a total of 12 frames. Coronal Cine-FLASH of the short axis scans were processed according to the modified Simpson rule at 1/3 (midventricular, Am) and 2/3 (apex) of the LV length [25]. LV volumes were determined via semi-automated delineation of the ventricle walls, using PMOD v.3.8 software (PMOD Technologies, Switzerland). To assess the myocardial infarction size, 0.3 µmol/g BW Gadolinium (Gadovist^®^ 1.0, Bayer AG, Zurich, Switzerland) was injected intravenously (tail vein) as a bolus. Subsequently, coronal short axis scans were performed from base to apex with a slice distance of 1 mm using the fast imaging with steady-state precision (FISP) sequence with the following parameter: field of view = 25 mm × 25 mm, matrix dimension = 96 × 96, slice thickness = 0.8 mm, flip angle = 15, repetition time = 5 ms, echo time = 2.5 ms, number of averages = 4. The volume of the infarcted area in the LV anterior wall was determined manually by drawing regions of interest delineating the affected region, and by dividing the infarct volume by LV end diastolic volume, as previously reported [26]. Cardiac output (CO) was calculated from the product of heart rate and stroke volume. Cardiac index (CI) was obtained by correcting CO for body weight. LV wall thickening was defined as the percentage of increase in wall thickening from end diastole to end systole, as previously reported [27].

### Serum levels of Progesterone and Testosterone

Blood samples were obtained by cardiac puncture and centrifuged at 10,000 g for 10 min. The resulting serum samples were snap frozen and stored at -80 °C until further analysis. Serum testosterone levels were measured using rodent enzyme-linked immunosorbent assay (ELISA) assays according to the manufacturer’s recommendations (Calbiotech, USA). The absorbance was determined at a wavelength of 450 nm and 570 nm for the pathlength correction using a Tecan infinite pro 200 reader (Tecan, Switzerland). A 4-parameter logistic regression algorithm was used to fit the standard curve. Mean sample hormone levels were calculated within a standard range of 0.1–18 ng/mL. Progesterone levels were assessed by liquid chromatography with tandem mass spectrometry (LC-MS/MS), as previously reported [28].

### Measurement of catecholamines in urine

Urine samples were used to assess catecholamine levels using an epinephrine ELISA kit (KA1877, Abnova Corperation, Taiwan). Extraction and subsequent ELISA was performed according to the manufacturer’s instructions. Absorbance was determined at a wavelength of 450 nm and 635 nm using a Tecan infinite pro 200 reader (Tecan, Switzerland). A 4-parameter logistic regression algorithm was used to fit the standard curve. Mean sample hormone levels were calculated for epinephrine in ng/mL in a standard range of 0.7–200 ng/mL.

### Statistical analysis

Data are presented as numbers and percentages for categorical variables or mean ± standard error of the mean (SEM) for continuous variables. The Shapiro-Wilk and Kolmogorov-Smirnov were employed to test for normality of distribution, and Levene’s test to evaluate homogeneity of variance. One-way analysis of variance with the Tukey’s post-hoc test or unpaired *t-*test were used for intragroup comparisons. For non-parametric data, the Kruskal-Wallis test with post-hoc Dunn’s multiple comparison test or Mann-Whitney test was employed, as appropriate. Frequency data analyses were performed by means of the Chi-Square tests. Two-way analysis of variance was performed to assess the interaction between age and sex hormones in males and females. The relationship between testosterone and cardiac sympathetic activity was evaluated using Spearman’s correlation coefficient. Outliers were excluded by the Grubbs’ test (α = 0.05). Statistical significance was set at *p* < 0.05. Statistical analyses were performed with IBM SPSS statistics v28.0 (IBM Corp. Armonk, NY), and figures were prepared with GraphPad Prism (v8.0, GraphPad Software, San Diego, CA).

## Results

### Sex differences in Infarct size and cardiac function

**Fig. 2:**
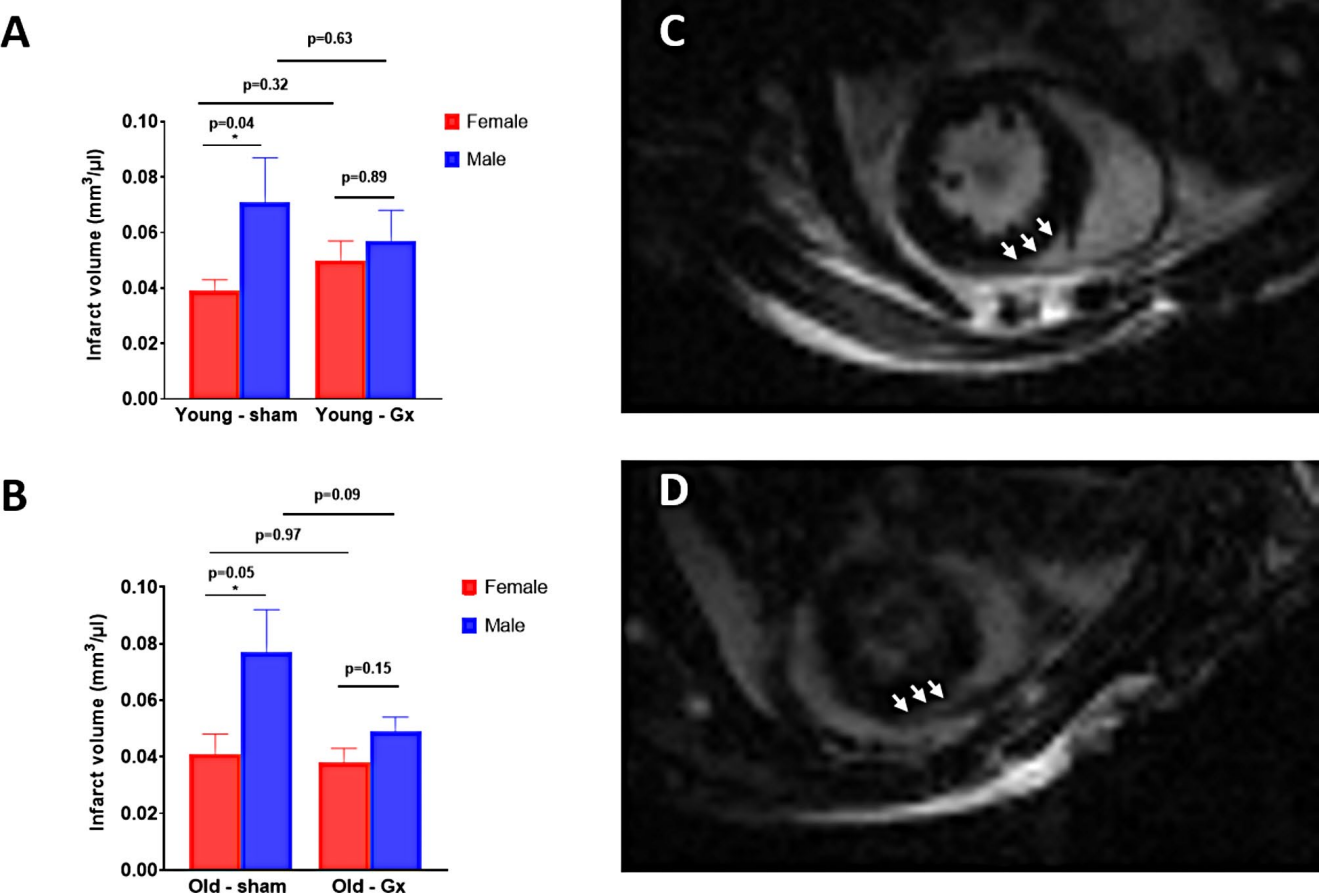
Infarct volume determined by cardiac magnetic resonance (CMR) imaging in FVB/N mice 24 h following myocardial injury. **A**. Young female vs. male animals at the age of 4–6 months, which were either gonadectomized (right) or sham operated (left). **B**. Old female vs. male animals at the age of 20–22 months, which were either gonadectomized (right) or sham operated (left). Representative late gadolinium enhancement (LGE) images showing the infarct area are depicted for the aged sham male (**C**) and aged sham female (**D**) heart. Infarct area is highlighted with white arrows. Abbreviations: ANOVA, analysis of variance; Gx, gonadectomized

An overall larger average infarct size was observed in sham male mice as compared to sham females (Fig. 2). This difference was most pronounced in young sham animals (Figs. 2A and 70.84 ± 16.27 vs. 39.23 ± 4.47 mm^3^/mL, *p* = 0.037), less pronounced in sham old animals (Fig. 2B, aged sham males vs. females, 76.92 ± 14.48 vs. 41.02 ± 7.38 mm^3^/mL, *p* = 0.048), and absent in gonadectomized animals (Figs. 2A and 56.78 ± 10.51 vs. 50.06 ± 7.11 mm^3^/mL, *p* = 0.89; Fig. 2B, aged males vs. aged females, 49.15 ± 5.30 vs. 37.71 ± 5.12 mm^3^/mL, *p* = 0.15). Gonadectomy did not significantly affect infarct volumes in any of the experimental groups. A two-way ANOVA for all animal groups (males and females) revealed the following main effects: sex: *p* = 0.003, age: *p* = 0.669; gonadectomy: *p* = 0.228, interaction p = NS. Similarly, a two-way ANOVA for all sham operated groups (males and females) revealed the following main effects: sex: *p* = 0.013, age: *p* = 0.763; interaction age*sex: *p* = 0.870. Representative images showing late gadolinium enhancement (LGE) in the infarct area are depicted for the aged male (Fig. 2C) and aged female (Fig. 2D) heart.

**Fig. 3:**
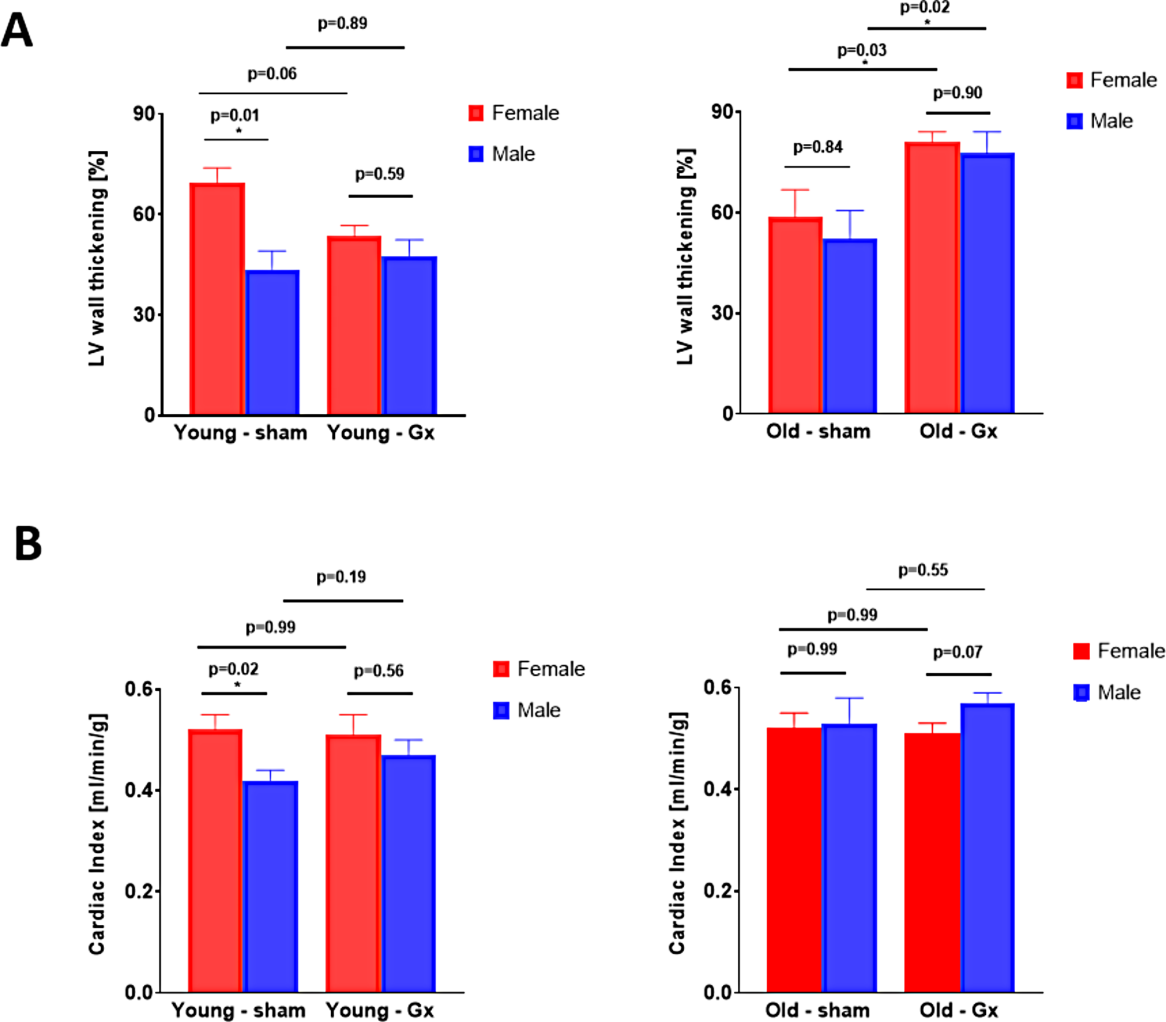
Left ventricular wall thickening and cardiac index assessed by cardiac magnetic resonance (CMR) imaging in FVB/N mice 24 h following myocardial injury. **A**. Left ventricular wall thickening in young and old female vs. male animals at the age of 4–6 and 20–22 months, respectively, which were either gonadectomized or sham operated, as indicated. **B**. Cardiac index in young and old female vs. male animals at the age of 4–6 and 20–22 months, respectively, which were either gonadectomized or sham operated, as indicated

Myocardial contractility following myocardial injury was impaired in young sham males as compared to young females, as evidenced by left-midventricular wall thickening (Fig. 3A, young sham males vs. females, 43.6 ± 5.5 vs. 69.5 ± 4.4%, *p* = 0.01) and cardiac indices (Fig. 3B, young sham males vs. females, 0.42 ± 0.02 vs. 0.52 ± 0.03 mL/min/g, *p* = 0.02). These sex differences vanished in Gx animals for both, LV wall thickening (Fig. 3A, young Gx males vs. females, 47.6 ± 4.8 vs. 53.4 ± 3.3%, *p* = 0.59) and cardiac indices (Fig. 3B, young Gx males vs. females, 0.47 ± 0.03 vs. 0.51 ± 0.04 mL/min/g, *p* = 0.56). A two-way ANOVA for all female groups revealed the following main effects: age: *p* = 0.964, gonadectomy: *p* = 0.782; interaction age*gonadectomy: *p* = 0.957, while the same ANOVA for all male groups provided the following main effects: age: *p* = 0.002, gonadectomy: *p* = 0.168; interaction age*gonadectomy: *p* = 0.971. Abbreviations: ANOVA, analysis of variance; Gx, gonadectomized; LV, left ventricular. While cardiac indices were not affected by gonadectomy in any of the groups (Fig. 3B), both ovariectomy and orchiectomy were associated with a heightened LV wall thickening following MI in old animals (Fig. 3A). No sex differences in myocardial contractility were detected in aged animals following myocardial injury.

A two-way ANOVA for all female groups revealed the following main effects: age: *p* = 0.074; gonadectomy: *p* = 0.488; interaction age*gonadectomy: *p* < 0.001, while the same ANOVA for all male groups showed the following main effects: age: *p* = 0.005; gonadectomy: *p* = 0.031; interaction age*gonadectomy: *p* = 0.112. Young sham females had a slightly lower body weight than age-matched males (*p* = 0.002, Supplemental Fig. 1), while no significant sex differences for body weight were observed in the other experimental groups. Resting heart rate did not differ between experimental groups (Supplemental Fig. 2).

### Myocardial sympathetic activity following myocardial injury is higher in males than in females

**Fig. 4:**
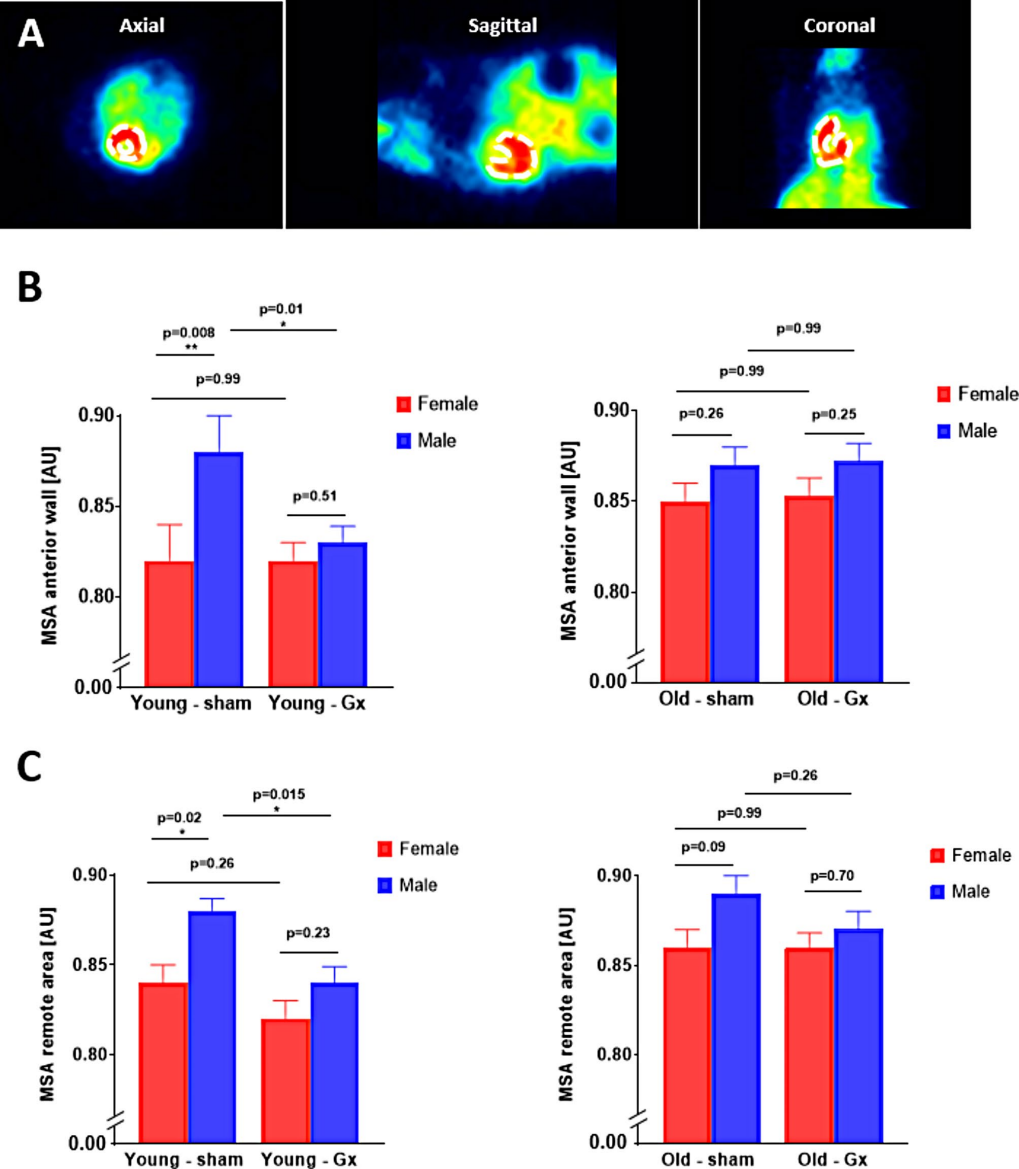
Myocardial sympathetic activity (MSA) assessed by [^11^C]mHED PET in an FVB/N mouse model of myocardial injury. **A**. Representative images of the mouse myocardium (highlighted by dashed line) 24 h following I/R injury and after intravenous [^11^C]mHED administration. **B**. Comparison of MSA at the anterior wall (infarct region) in young and old female and male animals that underwent gonadectomy (right) or sham surgery (left), respectively. **C**. Comparison of MSA at a remote region (non-infarct region) in young and old female and male animals that underwent gonadectomy (right) or sham surgery (left), respectively. Abbreviations: ANOVA, analysis of variance; Gx, gonadectomized; AU, arbitrary units

Representative images of the mouse myocardium 24 h following myocardial injury and after intravenous [^11^C]mHED administration are depicted in Fig. 4A. MSA was assessed within the LV anterior wall (infarct region) and revealed higher MSA in young males as compared to age-matched females (young males vs. young females, 0.88 ± 0.02 vs. 0.82 ± 0.02, *p* = 0.008 Fig. 4B). This sex difference was not observed in aged animals (aged males vs. aged females, 0.87 ± 0.01 vs. 0.85 ± 0.01, *p* = 0.26, Fig. 4B). MSA following myocardial injury was highest in young males, and was significantly reduced in the absence of testosterone (orchiectomy effect [Gx], *p* = 0.01, Fig. 4B). In contrast, estrogen depletion did not affect MSA in young females (ovariectomy effect [Gx], *p* = 0.99, Fig. 4B). Notably, female animals showed an age-dependent increase in MSA, which was absent in males (male aging effect, *p* = 0.253 and female aging effect, *p* = 0.011). A two-way ANOVA for all female groups revealed the following main effects: age: *p* = 0.011, gonadectomy: *p* = 0.892; interaction age*gonadectomy: *p* = 0.925, whereas the same ANOVA for all male groups revealed the following main effects: age: *p* = 0.235, gonadectomy: *p* = 0.013; interaction age*gonadectomy: *p* = 0.052.

To assess whether these observations were confined to the infarct region, a remote (non-infarcted) area was included in the analysis. MSA derived from the remote area revealed results comparable to those in the infarct region, pointing towards a global effect of myocardial injury on MSA (Fig. 4C). A two-way ANOVA for all female groups revealed the following main effects: age: *p* = 0.006, gonadectomy: *p* = 0.483; interaction age*gonadectomy: *p* = 0.568, whereas the same ANOVA for all male groups revealed the following main effects: age: *p* = 0.024, gonadectomy: *p* = 0.002; interaction age*gonadectomy: *p* = 0.328.

### Association between sex hormone serum levels and MSA

Testosterone levels were substantially reduced in orchiectomized animals (**Supplemental Fig. 3A**). In sham-operated males, no age-dependent change in serum testosterone levels was observed, suggesting that testosterone levels are preserved with age in male FVB/N mice (**Supplemental Fig. 3A**, young normal vs. aged normal, 11.7 ± 8.0 vs. 11.8 ± 5.2 nM, *p* = 1.00). Notably, orchiectomized males showed an age-dependent increase in serum testosterone levels, suggesting the activation of an alternative testosterone production pathway in aged gonadectomized males such as the progesterone-androstenedione-testosterone pathway [29–31] (**Supplemental Fig. 3A**, young gonadectomized vs. aged gonadectomized, 0.1 ± 0.03 vs. 0.6 ± 0.15 nM, *p* = 0.05). There was a significant and positive correlation between testosterone serum levels and MSA across all male study groups (**Supplemental Fig. 3B**, r = 0.660, *p* < 0.001). Due to the known challenges associated with the measurement of low serum estradiol concentrations in mice [32, 33], female gonadectomy was confirmed by significantly reduced progesterone levels in young females (**Supplemental Fig. 4A**, young sham vs. young gonadectomized, 11.2 ± 1.59 vs. 2.96 ± 0.44 nM, *p* < 0.001), which was not significant in aged females (**Supplemental Fig. 4B**, old sham vs. old gonadectomized, 5.86 ± 2.22 vs. 4.00 ± 1.96 nM, *p* = 0.218), potentially owing to an age-dependent decline in sex hormones with age in the sham-operated female cohort. Female gonadectomy was further corroborated by visually confirming the absence of gonads following euthanasia. There was no correlation between serum progesterone levels and MSA in females (data not shown). Testosterone levels were below the limit of quantification in most female FVB/N mice, preventing a robust analysis of its impact on MSA.

### Effect of gonadectomy on epinephrine levels

**Fig. 5:**
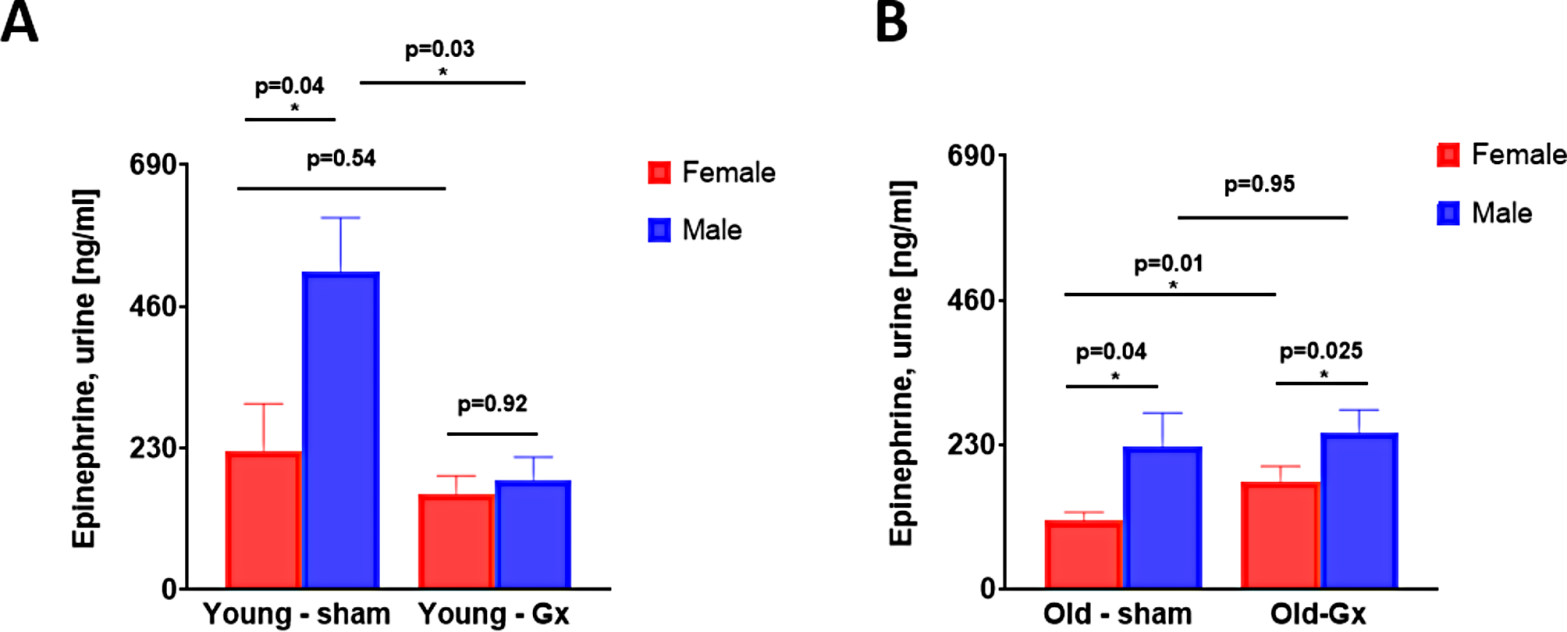
Epinephrine levels obtained from urine sample analyses in FVB/N mice subjected to myocardial injury. **A**. Young female vs. male animals at the age of 4–6 months, which underwent either gonadectomy (right) or sham surgery (left). **B**. Old female vs. male animals at the age of 20–22 months, which underwent either gonadectomy (Gx, right) or sham surgery (left). Abbreviations: ANOVA, analysis of variance; Gx, gonadectomized

Urine epinephrine levels 24 h post myocardial injury were significantly higher in sham-operated males than in females, independent of age (Fig. 5A, young males vs. young females, 517 ± 88 vs. 226 ± 77 ng/mL, *p* = 0.043; Fig. 5B, aged males vs. aged females, 226 ± 54 vs. 109 ± 13 ng/mL, *p* = 0.04). Orchiectomized young males exhibited significantly reduced urine epinephrine levels (Fig. 5A), whereas ovariectomized old females showed elevated urine epinephrine concentrations (Fig. 5B). In Gx animals, significant sex differences in epinephrine levels were only observed in the aged cohort (Fig. 5A, young males vs. young females, 178 ± 37 vs. 156 ± 29 ng/mL, *p* = 0.92; Fig. 5B, aged males vs. aged females, 249 ± 36 vs. 171 ± 24 ng/mL, *p* < 0.025, respectively). A two-way ANOVA for all female groups revealed the following main effects: age: *p* = 0.369, gonadectomy: *p* = 0.945; interaction age*gonadectomy: *p* = 0.247, whereas the same ANOVA for all male groups revealed the following main effects: age: *p* = 0.041, gonadectomy: *p* = 0.005; interaction age*gonadectomy: *p* = 0.002.

## Discussion

Contrary to our hypothesis, following myocardial injury, male animals in our in vivo imaging study had a larger infarct size, reduced myocardial contractility, and higher MSA, as assessed by [^11^C]mHED PET, than female animals. Both MSA and catecholamine levels were highest in young males following myocardial injury and were significantly reduced by testosterone depletion through orchiectomy. Accordingly, testosterone serum levels correlated positively with MSA. Sex differences in infarct size and myocardial contractility following myocardial injury disappeared in gonadectomized animals. In females, MSA post myocardial injury significantly increased with age, however, this age-dependent increase remained unaltered in ovariectomized mice. Our findings indicate that elevated testosterone levels in males are associated with MSA, which contributes to adverse early post-MI left ventricular remodeling. In contrast, females maintain stable sympathetic activity, and ovariectomy does not alter MSA in females, suggesting sex-specific mechanisms in post-MI cardiac remodeling. While our data confirm recent clinical data showing an age-dependent increase in sympathetic activity [18] as well as a more concentric remodeling following MI in women [34], our observations point towards a biological advantage of female sex in the early phase following ischemic myocardial injury – independent of female sex hormones.

Despite the growing awareness of sex and gender disparities in the patient’s trajectory following type I MI [4], mechanistic evidence explaining these differences is currently lacking. In fact, while activation of the sympathetic nervous system has been identified as a key player in many cardiac conditions including myocardial infarction [35], it is currently unclear how sex hormones affect MSA and cardiac remodeling following type I MI. While ovariectomy did not affect MSA in our study, an age-dependent increase in MSA was observed in females. This is an important observation, as it underscores the complexity of studying age-related changes in female MSA. Beyond an age-related decline in sex hormone levels, other potential factors that could contribute to an enhanced MSA in aged females may include changes in adrenergic receptor sensitivity, chronic low-grade inflammation, and alterations in autonomic balance [36–38]. While female sex hormones appear to have a limited impact on MSA in aged mice, other age-related physiological changes may drive the increase in sympathetic tone.

During an acute MI, norepinephrine is increasingly released from sympathetic nerve terminals, promoting sympathetic hyperactivity and subsequent cardiac remodeling [39]. Notably, there is solid evidence that sympathetic hyperactivity is not only associated with progression of atherosclerosis [40], but also with the extent of myocardial damage and fibrosis formation after MI. Indeed, McAlpine and coworkers reported an association between persistently high catecholamine levels post MI and ventricular tachycardia, acute heart failure, or death [41]. Similarly, a protracted state of sympathetic hyperactivity was found after uncomplicated MI in a small predominantly male population, which, in turn, has implications for the extent of myocardial damage and late mortality [42]. Our study now provides further insight into these associations by demonstrating that (1) there are significant sex differences in MSA as well as in the extend of myocardial damage in the early phase following myocardial injury and (2) that testosterone is linked to these sex differences. While ovariectomy did not influence MSA or urinary epinephrine levels, orchiectomy in young males was associated with changes in both MSA and urinary epinephrine. This supports the hypothesis that testosterone contributes to enhanced myocardial adrenergic signaling.

Associations between testosterone levels and various determinants of the cardiovascular system have been described in experimental models [43]. Testosterone treatment of rabbits showed anti-arrhythmic properties by reducing the basal action potential duration to 90% repolarization (APD_90_) in rabbits [44]. Similarly, testosterone was found to shorten ventricular repolarization duration in mice, potentially owing to an increased expression of Kv1.5 potassium channels [45]. It was further suggested that testosterone may provide cardio-protection in rats by activating ATP-sensitive mitochondrial potassium channels, thereby stabilizing the mitochondrion in ischemic cardiomyocytes [46]. These studies, however, did not account for sympathetic activity, nor did the authors use in vivo models of (I/R) injury. In our study, testosterone was correlated with an enhanced MSA on [^11^C]mHED PET. Given the well-established mechanism of myocardial uptake and retention of [^11^C]mHED, which is mediated by the norepinephrine transporter (NET), our findings suggest that plasma testosterone levels influence the modulation of NET expression or activity at sympathetic nerve terminals (Fig. 1) – a hypothesis supported by previous studies showing that androgens can regulate neurotransmitter transporter expression [19, 47, 48]. In the absence of cardiac sympathetic denervation, [^11^C]mHED retention is inversely correlated with MSA, and this inverse relationship arises because sympathetic activation leads to increased norepinephrine release, reducing the reuptake of both norepinephrine and [^11^C]mHED through NET into the presynaptic nerve terminal [19, 20, 49]. Therefore, higher levels of sympathetic activation result in decreased [^11^C]mHED retention, ultimately reflecting reduced NET-mediated reuptake under conditions of heightened sympathetic tone. While our findings hint toward a link between testosterone and NET, further studies are warranted to establish causality. Indeed, further experiments that directly investigate the pathways through which testosterone modulates sympathetic activity, including the histological assessment of NET expression levels, may further shed light on the mechanistic understanding of the observed link between testosterone and MSA. In the present study, testosterone plasma concentrations were significantly correlated with MSA, however, it should be noted that the correlation was accentuated by a few individual males with relatively high testosterone. A sensitivity analysis revealed that while the correlation between testosterone levels and MSA persisted, its strength varied depending on data subsets, thus warranting a cautious interpretation and underscoring the need for further studies to confirm causality.

The observed age-related discrepancy in the relation between testosterone levels and MSA likely stems from multifactorial mechanisms. One potential explanation involves the compensatory production of extra-gonadal testosterone in aged gonadectomized males, as indicated by our data (Supplemental Fig. 3). While the testosterone levels in these animals were lower than those observed in sham-operated males, they were still associated with an increase in MSA. This supports the concept that relatively low circulating levels of testosterone may be sufficient to affect sympathetic activity, differentiating the physiological effects of reduced testosterone from those of complete androgen depletion. The maintenance of sympathetic tone in aged animals may therefore be partially attributed to these residual androgen levels, despite the absence of gonadal testosterone, which highlights the complexity of hormonal regulation in the aging heart.

Although experimental studies investigating the effect of testosterone on infarct size are limited, most of them showed an infarct size-limiting effect of testosterone treatment [50]. However, the vast majority of these studies were conducted in young rats, and there is currently a lack of studies assessing the interaction effect of age and testosterone on infarct size. Further, most of these studies were performed on the isolated perfused heart or isolated cells and may not appropriately reflect the in vivo situation. For example, testosterone treatment was cardioprotective via α1-adrenoceptor stimulation in isolated perfused hearts that were subjected to myocardial injury [51]. In another in vitro study with isolated ventricular myocytes, testosterone treatment provided improved myocyte viability following myocardial injury by enhancing the expression of heat shock protein 70 [52]. It should be noted however, that these studies used tissues derived from relatively young male rodents and did not account for potential age and sex differences. In contrast, we have performed myocardial injury in young and aged mice of both sexes and determined the infarct size by non-invasive in vivo imaging. Following 30 min ischemia and a 24 h-reperfusion period, we found a trend towards larger infarct sizes in aged males with intact gonads, as compared to their female counterparts. We have used FVB/N mice, a strain with high physiological testosterone levels resembling normal testosterone levels in men, as confirmed by ELISA experiments. Of note, infarct size and LV remodeling post-MI are inherently linked to the time point at which measurements are taken, and previous studies have shown that the effects of testosterone on cardiac remodeling can vary depending on the acute vs. chronic phases of myocardial infarction [53]. Further, the removal of endogenous testosterone through gonadectomy may have triggered adaptive mechanisms that attenuated the potential cardioprotective impact of a reduced baseline sympathetic tone. For example, testosterone depletion can lead to alterations in other hormone levels, such as adrenal androgens or stress hormones like cortisol (corticosterone in mice), which might in turn may fuel the inflammatory response following MI [54–56]. Other factors, such as differences in adrenergic receptor expression and inter-individual variations in inflammatory response could also play a role in influencing infarct size. Further analysis focusing on markers of adrenergic signaling and inflammatory response could provide more insight into these potential confounders [57]. Notably, a recent study conducted in a mouse model of myocardial sympathetic denervation and MI revealed that myocardial inflammation was attenuated, and cardiac function was improved, in mice with local sympathetic denervation [39]. These findings suggest that cardiac sympathetic activation triggers an enhanced myocardial inflammatory response after an ischemic injury. Along this line, Levick et al. found that sympathectomy normalized myocardial levels of interferon (IFN)-gamma, interleukin-6 (IL-6) and interleukin-10 (IL-10) in hypertensive rats [58]. While our study did not assess myocardial inflammation, our results point toward a detrimental role of testosterone treatment in elderly males, which may be accompanied by an enhanced sympathetic drive following MI. Further, given that testosterone treatment is being increasingly used in elderly men with hypogonadism and erectile dysfunction [59, 60], our findings highlight the importance of well-designed prospective clinical trials accounting for the effects of testosterone replacement therapy on the sympathetic nervous system. To do so, we advocate the use of non-invasive assessment of MSA as an adjunct to the current standard of care in cardiovascular trials for testosterone replacement therapy. The latter may help to identify subpopulations that benefit most from testosterone replacement therapy.

While testosterone is traditionally regarded as a male sex hormone, it also plays a role in female physiology, albeit at much lower concentrations. In our study, the testosterone levels in most female FVB/N mice were below the limit of quantification, preventing a robust analysis of its impact on MSA. However, it is important to acknowledge that even low levels of testosterone may exert physiological effects in females, particularly in the context of cardiovascular function. Progesterone, a precursor to testosterone, may serve as an indirect regulator of MSA through its conversion to androgens, highlighting the potential for cross-talk between these hormones. While our focus was on the pronounced effects of testosterone in males, future studies could explore more sensitive methods to assess the role of low-level androgens in females, potentially providing a more comprehensive understanding of how sex hormones influence cardiac remodeling and sympathetic activity across sexes.

Our study has several limitations. First, our study was designed to detect a large effect size. As such, in between group differences of a small effect size might not have been detected by our study. Further, while we acknowledge the limitation of potentially missing out on basal age- and sex-differences, we would like to highlight that our dataset includes a wide range of parameters in a total of eight experimental groups, thus providing valuable insight into how age, sex and gonadectomy are linked to MSA and cardiac function in the specific context of myocardial injury. Importantly, the focus on post-MI animals allowed us to assess how these variables impact recovery and LV remodeling. Future studies could explore baseline differences between non-MI and post-MI animals – with the aim to put the present findings into a broader context. Second, while our study suggests a biological advantage of female sex post MI, sex differences in inflammation and/or atherosclerosis development, such as the higher prevalence of non-obstructive CAD in women, have not been adequately captured by our experimental model. Third, our animal model does not reflect the complexity of human atherosclerotic disease, and age-dependent declines in female sex hormones in mice are not equivalent to menopause in women [61, 62]. Indeed, while we show a significant decline in female sex hormone levels in old females, our study does not adequately address the questions of “timing and duration” of that decline. Despite this limitation, the broader implications of age-related hormonal changes on MSA and cardiac outcomes were captured in an MI model that has been rigorously validated over the past decades, unveiling a series of translational observations, which include physiological, cellular and molecular adaptations [12, 63]. Finally, using [^18^F]DOPA PET, we have previously shown that healthy older women display a higher sympathetic activity of the LV apex than younger women or men of all ages [18]. Conversely, in a previous clinical study encompassing 72 elderly subjects, MSNA was higher in men than in women under baseline conditions, while the magnitude of MSNA following MI was greater in women [16]. While both methods are limited in their ability to reflect MSA, the question arises whether the relative change of sympathetic changes following a cardiovascular event may be a superior risk marker than the absolute level of sympathetic tone. The lack of data on sympathetic tone before and after myocardial injury precludes any conclusions on the relative change of sympathetic activation in our study, which may be considered a study limitation. Indeed, considering that sympathetic hyperactivation has been described as key pathological hallmark of Takotsubo cardiomyopathy, which is predominantly observed in women (89.8%) [64], this concept warrants further investigation in longitudinal studies.

In conclusion, we provide experimental evidence that testosterone contributes to an enhanced MSA and an increased infarct size following myocardial injury in male mice. Given the broad use of testosterone replacement therapy in elderly men with hypogonadism and erectile dysfunction, our results imply that the treatment of elderly men with testosterone merits careful consideration. Further, we show that young females are protected from myocardial injury relative to age-matched males, as evidenced by their reduced sympathetic tone, their smaller infarct size as well as their superior cardiac contractility. Clinical studies are required to confirm these findings in humans.

## Electronic supplementary material

Below is the link to the electronic supplementary material.

Supplementary Material 1

## Data Availability

No datasets were generated or analysed during the current study.

## Declarations

### Implications for patient care

Testosterone replacement therapy in elderly men (e.g. with hypogonadism or erectile dysfunction) merits careful consideration of potential adverse outcomes following myocardial infarction.
