# Supplementary material for: Age- and sex-specific differences in myocardial sympathetic tone and left ventricular remodeling following myocardial injury
Source: Biol Sex Differ. 2025 Jan 16;16:2. doi: 10.1186/s13293-024-00673-5 (PMC11737239; doi:10.1186/s13293-024-00673-5)
Supplement: Supplementary file 1 — Supplementary Material 1 [file 13293_2024_673_MOESM1_ESM.pdf]

# Supplemental Figure 1

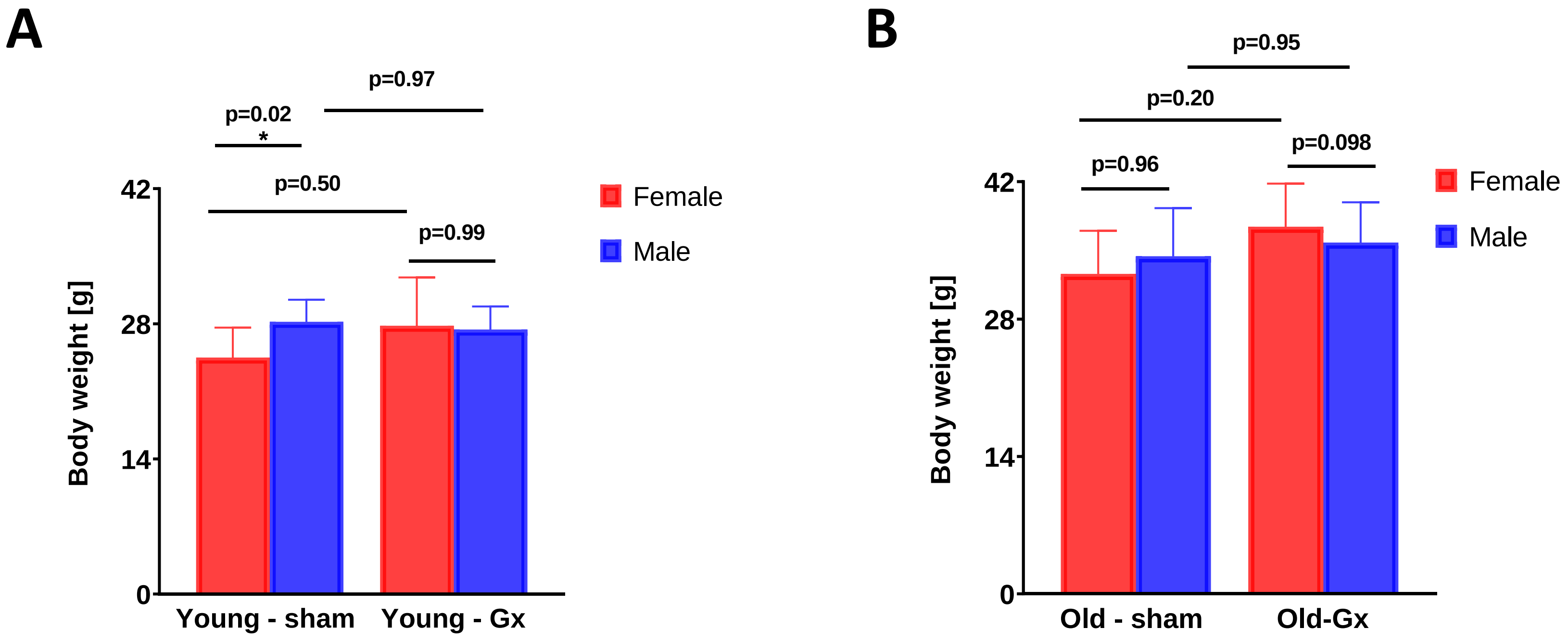


**Supplemental Fig 1**: Body weight of FVB/N mice 24 hours following myocardial injury. **A**. Young female vs. male animals at the age of 4-6 months, which underwent either gonadectomy (right) or sham surgery (left). **B**. Old female vs. male animals at the age of 20 months, which underwent either gonadectomy (right) or sham surgery (left). ANOVA for all female groups revealed the following main effects: age: $p<0.001$, gonadectomy: $p=0.2$; interaction age*gonadectomy: $p=0.949$. ANOVA for all male groups revealed the following main effects: age: $p<0.001$, gonadectomy: $p=0.136$; interaction age*gonadectomy: $p=0.353$. Abbreviations: ANOVA, analysis of variance; Gx, gonadectomized.

# Supplemental Figure 2

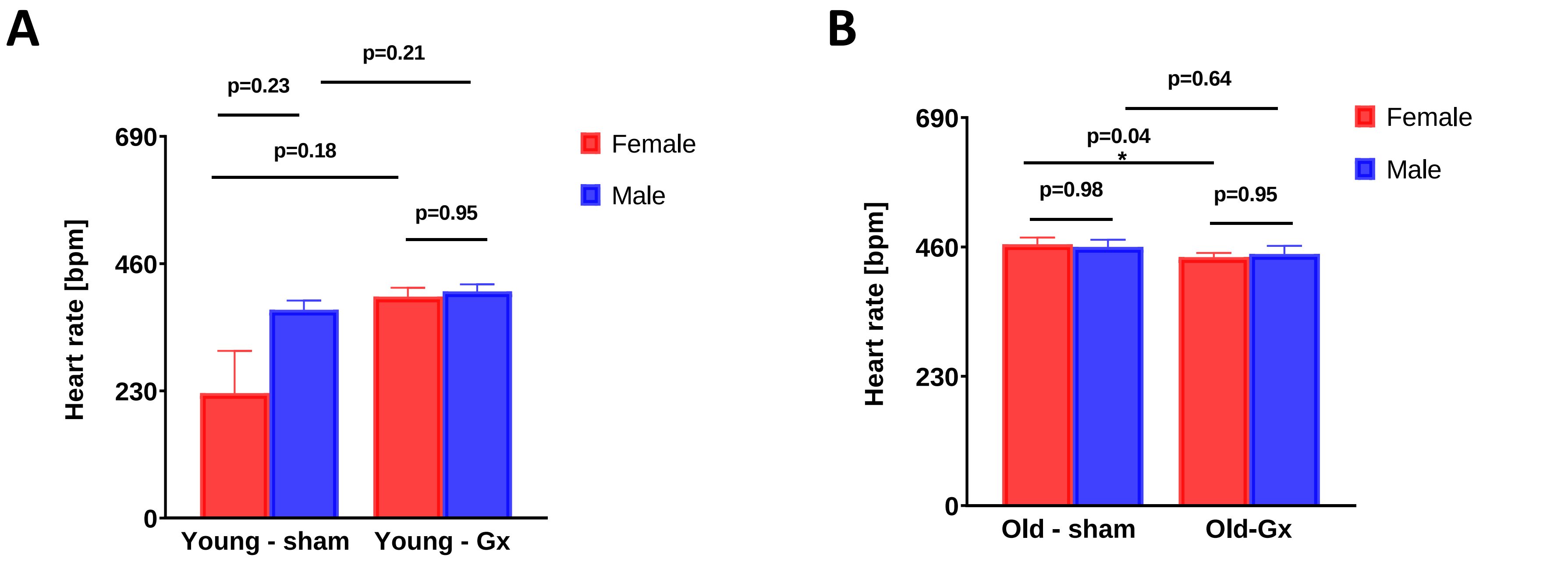


**Supplemental Fig 2**: Heart rate of FVB/N mice 24 hours following myocardial injury. **A**. Young female vs. male animals at the age of 4-6 months, which underwent either gonadectomy (right) or sham surgery (left). **B**. Old female vs. male animals at the age of 20 months, which underwent either gonadectomy (right) or sham surgery (left). ANOVA for all female groups revealed the following main effects: $p<0.001$, gonadectomy: $p=0.983$; interaction age*gonadectomy: $p=0.113$. ANOVA for all male groups revealed the following main effects: age: $p<0.001$, gonadectomy: $p=0.459$; interaction age*gonadectomy: $p=0.116$. Abbreviations: ANOVA, analysis of variance; bpm, beats per minute; Gx, gonadectomized.

# Supplemental Figure 3

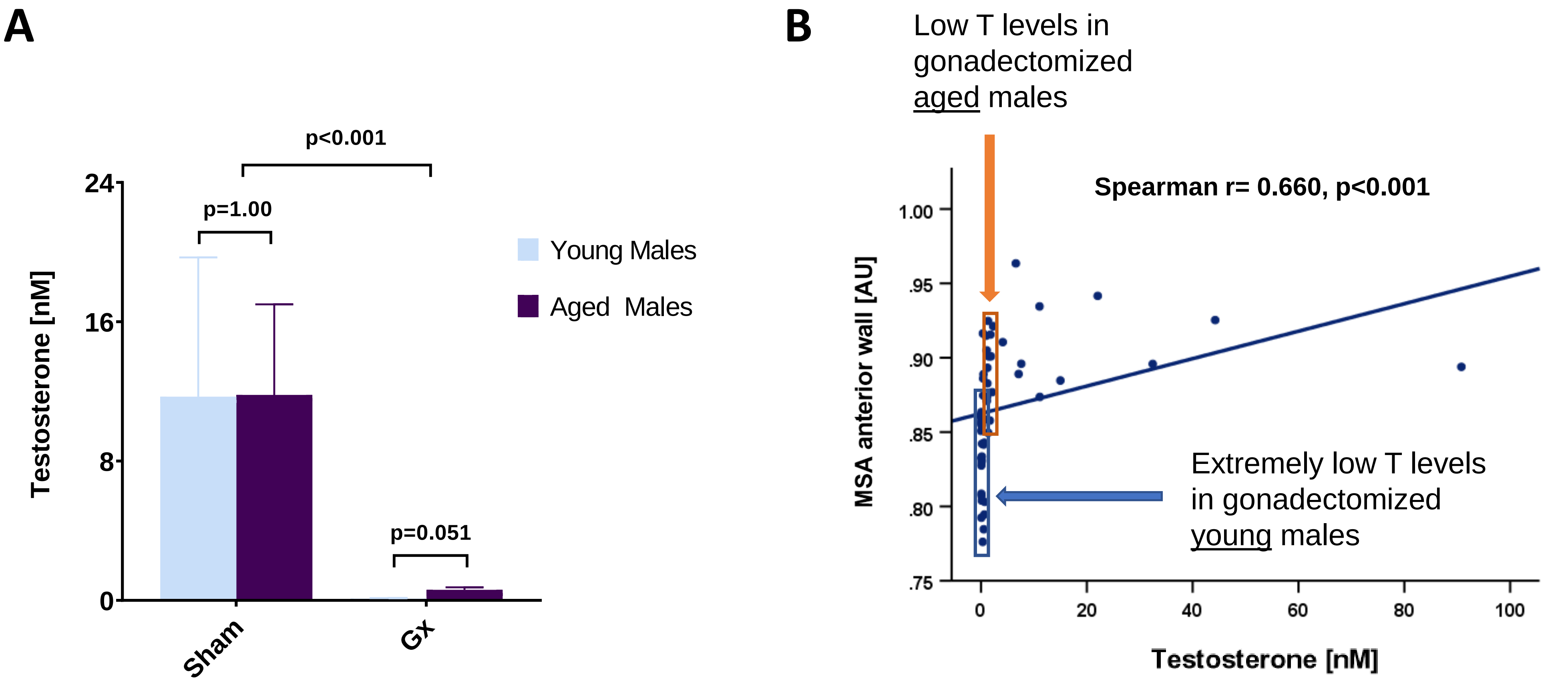


**Supplemental Fig 3**: Serum testosterone levels and correlation of myocardial sympathetic activity (MSA) with plasma testosterone in FVBN mice 24 hours following myocardial injury. **A**. Young vs. aged males that were either gonadectomized (right) or sham operated (left). **B**. Correlation between myocardial sympathetic activity by [$^{11}$C]mHED PET and analysis of serum testosterone level 24 hours following myocardial injury. Abbreviations: AU, arbitrary units; T, testosterone; Gx, gonadectomized.

# Supplemental Figure 4

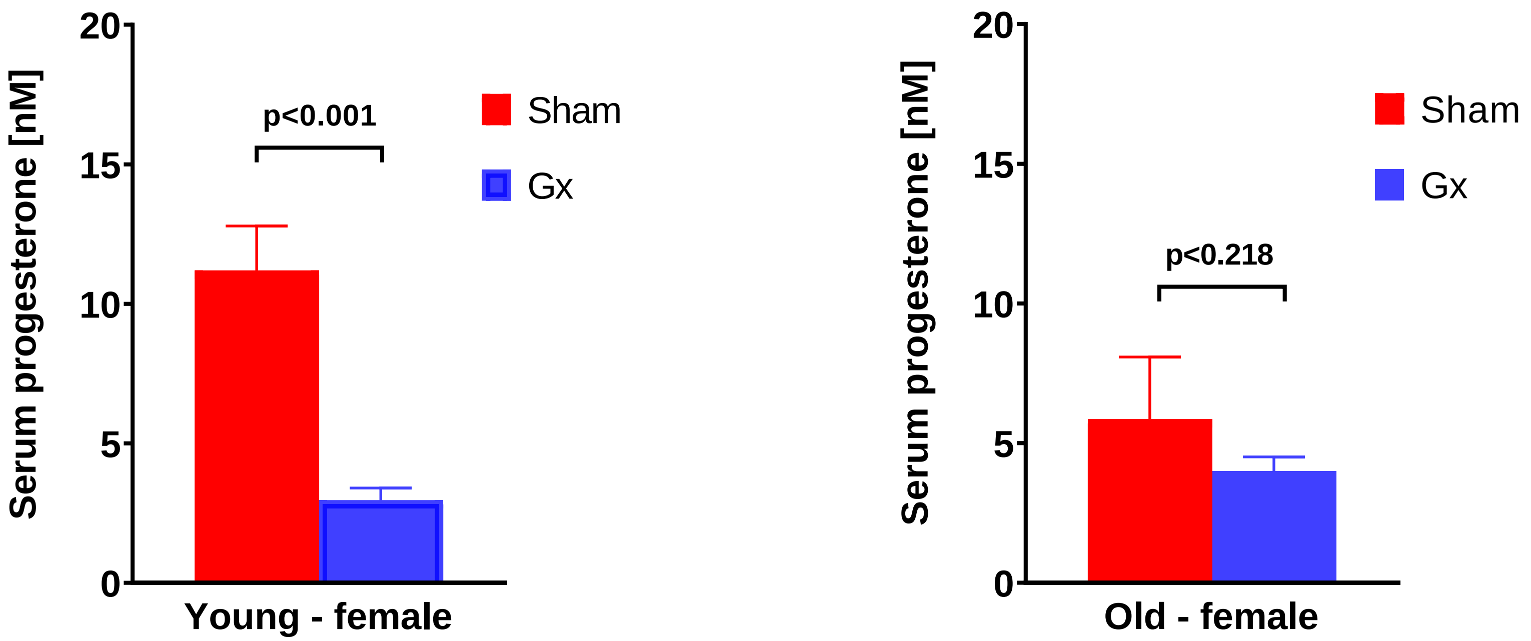


**Supplemental Fig 4:** Serum progesterone levels in female FVBN mice 24 h following myocardial injury. **A.** Sham operated vs. gonadectomized young and old female animals, respective.
